# Does attachment to God matter? Role of spiritual attachment in mental health through self-forgiveness: lessons from Turkish college sample

**DOI:** 10.3389/fpsyg.2025.1603654

**Published:** 2025-06-04

**Authors:** Fatih Aydın

**Affiliations:** Department of Counseling and Guidance, Sivas Cumhuriyet University, Sivas, Türkiye

**Keywords:** spiritual attachment, flourishing, self-forgiveness, mental health, mediation

## Abstract

**Background:**

The present study examines the role of spiritual attachment and self-forgiveness in flourishing among college students. A mediation model was tested in which flourishing served as the outcome variable, spiritual attachment as the independent variable, and self-forgiveness as the mediator.

**Methods:**

The sample consists of 311 (72.3%) females and 119 (27.7%) males, totaling 430 volunteered college students from 58 colleges and 18 faculties in Türkiye. The average age was 21.52 (3.49) for the total sample. Demographic Information Form, the Flourishing Scale, the Muslim Spiritual Attachment Scale, and the Self-Forgiveness Dual-Process Scale were utilized for data collection. A mediation model was tested based on the distinct subscales of self-forgiveness: value reorientation and esteem restoration.

**Results:**

The findings demonstrated that spiritual attachment significantly predicts flourishing among college students. Furthermore, both value reorientation and esteem restoration significantly predicted flourishing and played a mediator role in the relationship between spiritual attachment and flourishing.

**Conclusion:**

Together, it can be concluded that greater spiritual attachment may directly and indirectly (via self-forgiveness) contribute to better mental functioning in life. The present study promises some valuable information for practitioners in the field on the role of spiritual attachment and self-forgiveness in the mental health of emerging adults.

## Introduction

Flourishing is one of the latest concepts in wellbeing research. It is considered both a synonym and tangible evidence of mental health (Huppert, 2009; Ryff and Singer, 1998). Flourishing refers to optimal functioning, encompassing wellness, productivity, growth, and resilience (Fredrickson and Losada, 2005). This definition primarily draws on the concept of eudaimonia, as framed by Aristotle, which defines a good life as one in which people realize their potential, engage with life meaningfully, and find their purpose (Antonovsky, 1993; Ryan and Deci, 2001). Eudaimonia represents the objective “good” and is sought after because its essence is valuable (Rasmussen, 1999). On the other hand, the hedonic perspective of flourishing focuses more on subjective indicators of positive mental health: pleasure and the avoidance of pain (Diener et al., 1999; Diener, 1984). From this viewpoint, wellbeing is understood through positive affect and life satisfaction, whereby individuals seek experiences that maximize pleasurable feelings and minimize discomfort. Some well-established definitions merge these distinct perspectives, suggesting that flourishing can be achieved by functioning effectively and feeling good (Huppert and So, 2013; Cabrera and Donaldson, 2024; Keyes, 2002; Seligman, 2002). Pioneers in the subject have also identified key attributes that flourishers often credit for their wellbeing, including competence, self-esteem, accomplishment, optimism, physical health, financial security, positive mindset, mastery, autonomy, vitality, emotional stability, and virtue (Agenor et al., 2017; Huppert, 2009; Ryan et al., 2008; Seligman, 2011; VanderWeele, 2017). In addition, recent conceptualizations of flourishing go beyond just psychological health and include social wellbeing (Diener et al., 2010; Keyes, 2015). This highlights that flourishing also involves fulfilling social roles and obligations, as individuals are part of a broader society. People flourish when they view society as comprehensible, feel that it is a place where they can grow, feel a sense of belonging and acceptance, find it reasonable, and see themselves as meaningful contributors (Keyes, 2002).

Taken together, life from a flourisher’s perspective seems perfect: it might be the ultimate state of the human psyche. As stressed by Huppert and So (2013), it is not the absence of mental disorders; it is the opposite. However, people might face life challenges and psychological issues that prevent them from functioning effectively and feeling good. Keyes (2002) points out that languishers, unlike flourishers, often feel hopeless and desperate and describe their lives as empty or hollow. Research indicates that languishing is associated with a series of unfavorable mental health outcomes, including depression and anxiety (Eisele, 2020), suicidal thoughts and behaviors (Oh et al., 2024), problematic internet use, cyberbullying, and cyber victimization (Law et al., 2020), substance and alcohol use (McGaffin et al., 2015), low job satisfaction and burnout (Capone and Petrillo, 2020), higher school dropout rates (Andersen et al., 2021), and so on. Therefore, it is crucial for individuals of all ages to avoid these adverse conditions associated with languishing and promote flourishing.

In the Islamic sense, flourishing can be attained through practicing activities and lifestyle defined by the divine law, which is based on the messages of the Qur’an and the sunnah (traditions and practices drawn from the life of the Prophet Mohammad) (Fadel, 2022). Therefore, it is not a subjective good, but it can be built upon the objective doctrine of the religion. Often referred to as sharia, these rules guide individuals on how to live a proper and meaningful life (Joshanloo, 2017). Scholars argue that many quotes from the Qur’an encourage individuals to adopt positive attitudes and behaviors that lead to happiness and wellbeing (Omais and dos Santos, 2022). The suggestions provided by the sacred scriptures are comprehensive and holistic, including spiritual, mind–body, and social-environmental aspects (Ali, 2025). For instance, being close to God, taking good care of the body and mind, being kind to others, helping people in need, protecting orphans, treating women and the elderly well, and so on. In Türkiye, the state does not officially endorse sharia or Islamic law. Although most of the population is Muslim, the degree to which they apply sharia in their lifestyle varies. Instead, Turkish culture comprises Western and Eastern elements, creating a unique cultural harmony. Hence, it is possible to see the reflections of both Western and Eastern flourishing concepts in Türkiye.

Promoting better mental health in emerging adults is vital as they are in a period where they acquire undergraduate education, master vocational skills, build a career and daily routines, and engage in a romantic relationship that might evolve into a lifetime partnership. There seem to be many tasks emerging adults need to fulfill and many challenges to overcome. Thus, research stresses that college students live through both flourishing and languishing. Still, they are at risk of languishing, as they must deal with many uncertainties about the future, withstand practical hardships, cope with social isolation, and manage through overwhelming times in academic struggles (Knoesen and Naudé, 2018). In a comprehensive study with a large sample size, 36.24 percent of the 115,225 college students living in the USA were reported as flourishing (Oh, 2023). A similar picture is also accurate in the Healthy Minds Study 2023–2024 Data Report, where the mental health indicators were explored in the sample of 104,729 American college students (Healthy Minds Network, 2024). The results demonstrated that 38 percent of the sample were flourishing, 19 percent showed severe, and 38 percent had moderate to severe depressive symptoms. These statistics show that the mental health of approximately 60 percent of college students may be at stake. There might be many reasons behind the low rate of flourishing among college students, including unmet expectations and failure to connect life events purposefully (Shin, 2023), loneliness and social rejection (Ozawa-de Silva, 2020), and academic stress (Córdova Olivera et al., 2023). A growing body of research investigates the protective factors of flourishing, such as creativity (Conner et al., 2018), social support (Schotanus-Dijkstra et al., 2016), and positive experiences from various domains (VanderWeele, 2017) among college students. However, the limited number of studies on the subject highlights the need for further research. Therefore, the present study aims to uncover potential factors contributing to better mental health in the college sample. Specifically, flourishing was adopted as a mental health indicator, instead of other theoretical constructs such as life satisfaction and subjective wellbeing, and psychological symptoms such as depression and anxiety, because many psychologists refer to it as the ultimate state of wellbeing: a holistic model of human mental functioning.

## The current study and the development of the hypotheses

### Spiritual attachment as a predictor

Attachment represents the emotional bond developed between parents and infants during infancy (Bowlby, 1969). Depending on the availability of the parents, infants develop attachment styles to deal with environmental threats and hazards. If the caregivers are present when the infants look for them as an attachment figure to cope with the distress created by natural cues of danger, they build a secure attachment style (Ainsworth, 1979). Therefore, parents function as a “safe haven” and “secure base” for them to explore the environment freely and take shelter as threats become apparent (Bowlby, 1973). By internalizing these early relationships and the later interpersonal experiences, people develop internal working models that define how to be with a person (Bowlby, 1969). These models then become stable and turn into a trait-like feature. Following the leading efforts put by Bowlby (1969, 1973) and Ainsworth (1979, 1985), researchers also applied the same model to familial and romantic relationships (Hazan and Shaver, 1987; Proctor et al., 2009; Kirkpatrick, 1995). The evidence frequently indicates that individuals with a secure attachment style cope more effectively with stressful situations (Howard and Medway, 2004), exhibit fewer psychological symptoms of anxiety and depression (Beatson and Taryan, 2003; Kerns and Brumariu, 2014), have a lower likelihood of self-harming and engaging in disruptive behaviors (Grocutt, 2009), and demonstrate a higher level of wellbeing (Karreman and Vingerhoets, 2012). Hence, attachment style plays a key role in how people overcome life stressors and how happy they are.

**Figure 1:**
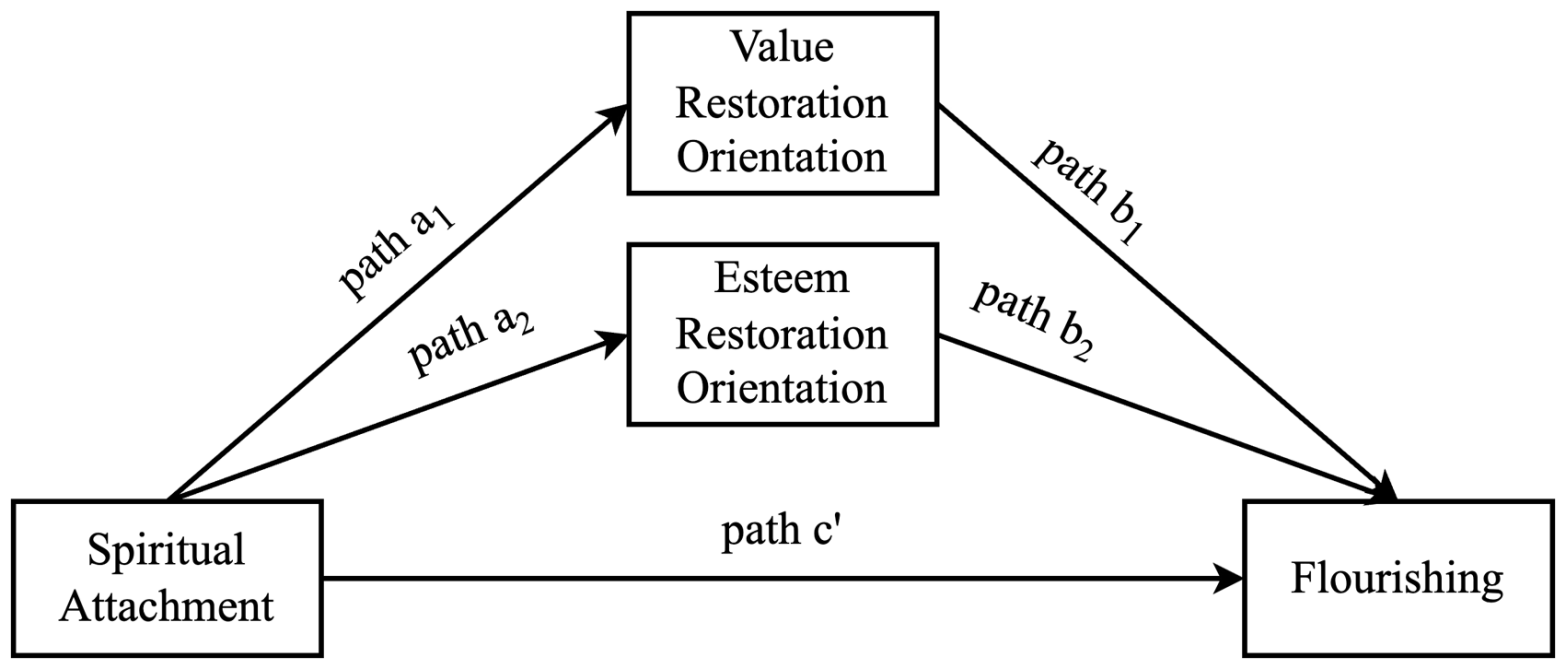
Hypothesized mediation model.

Similarly, Kirkpatrick and his colleagues applied the Attachment Theory to the spiritual sense of attachment to God (Kirkpatrick, 1992; Kirkpatrick and Shaver, 1992). Scholars argue that attachment to the parents, or humans in general, is associated with the attachment to the Sacred (Hall et al., 2009; Kirkpatrick, 2005). Like the attachment figures as humans, God is a well-suited attachment figure who guides and protects His servants and is seen as a safe haven and secure base for believers (Granqvist and Kirkpatrick, 2013). There were two assertions behind this conceptualization: the compensation and correspondence hypotheses. The compensation hypothesis suggests that individuals who had adverse childhood experiences, therefore, developed an insecure attachment to their caregivers, attached to God as a substitute to ease the feeling of distress, fulfill the need for love, and feel protected (Kirkpatrick, 1998). On the other hand, the correspondence hypothesis relies on the idea that attachment to God reflects the internal working models mentioned earlier. Therefore, the link between parental and spiritual attachment lies in the shared basis of the cognitive structure of relating to others (Figure 1).

A quote from the Qur’an draws attention to the close bond between God and His servants: “We are nearer to him than his jugular vein” (The Holy Quran, 2019; Surah Qâf, Verse 16–17, pp. 518). In Turkish culture, where Islamic philosophy gave rise to the Sufi tradition under the leadership of Mevlana Celaladdin Rumî, there is a central teaching that attachment to the objects of the world does not bring happiness; instead, being close to God does (Yalçınkaya, 2024). Therefore, in his poems, Mevlana seems to long to die, which he calls his “wedding night,” şeb-i arûs, where he would finally be able to meet the most beloved. Scholars have pointed out that Muslims always feel attached to God, and there are stages of attachment (Latifa et al., 2019). In the Sufi tradition, the spiritual journey is described through four fundamental stages: *sharia* (external religious law), *tariqa* (the inner spiritual path), *maʿrifa* (mystical knowledge), and *haqiqa* (ultimate truth). Sharia governs outward actions; tariqa involves self-purification and discipline; maʿrifa is intuitive knowledge of God; and haqiqa is the realization of divine reality (Özdemir, 2021). These stages reflect a movement from external observance to inward realization. Individuals develop a greater attachment through these stages by worshiping God and dedicating themselves to knowing Him better. In Türkiye, both Western and Eastern concepts of spiritual attachment have their cultural marks, as Turkish society is a part of both blocs.

In parallel with the parental attachment research, spiritual attachment serves as a protective factor. It helps cope effectively with stressors such as being hospitalized for serious diseases (Cassibba et al., 2014), fighting against behavioral problems such as alcohol use (Hernandez et al., 2010), and promotes better psychological adjustment (Miner, 2009). Likewise, emerging adults also have life stressors and try to deal with them spiritually (Sira et al., 2020). Therefore, it is thought that spiritual attachment might also be related to the flourishing of college students.

*H1*. Spiritual attachment has a positive direct effect on flourishing.

### Self-forgiveness as mediator

One of the spiritual concepts, self-forgiveness, plays a key role in mental health. It refers to “a willingness to abandon self–resentment in the face of one’s own acknowledged objective wrong while fostering compassion, generosity, and love toward oneself” (Enright, 1996, p. 115). Therefore, it is the process by which individuals uncover their wrongdoings, acknowledge their mistakes, take responsibility for rectifying them, try to empathize with themselves and understand that everyone commits offenses, develop self-awareness and compassion, and find meaning in what they experience (Webb et al., 2017). By forgiving themselves, individuals realize their self-worth and become aware that it dissociates from the offenses they have committed (Holmgren, 1998). Therefore, self-forgiveness enables individuals to discover value and purify themselves from self-directed hatred (Hall and Fincham, 2005). Furthermore, it involves esteem and value restoration (Griffin et al., 2018). Esteem restoration refers to the process through which individuals seek to regain a sense of self-worth and integrity after failing to uphold their moral or social standards (Griffin et al., 2024). On the other hand, value restoration encompasses an individual’s reaffirmation and recommitment to their core values and moral beliefs after a moral failing.

Similar concepts in Islamic literature align with the Western definition of self-forgiveness. The Quranic perspective is deeply ingrained in the fact that Allah has mercy (Rahmah): He forgives all sins except associating partners with Him, encourages His servants to ask for His mercy genuinely, and urges them to be hopeful for it (Rassool, 2024). Knowing God’s mercy, individuals engage in self-audit (Muhasabah), which involves acknowledging their past offenses, becoming aware of the sinful nature of the acts, and building self-awareness (Baharudin et al., 2019). Self-audit generally leads to repentance (Tawbah) and asking for forgiveness (Istighfar). Repentance and asking for forgiveness are processes in which people actively engage by recognizing their wrongdoing, feeling remorse, purifying the heart, turning themselves to divine law, changing wrong behaviors, and trying to repair the damages they have caused others (Toscano, 2022). In line with this, Robinson-Edwards (2022) states that self-forgiveness requires individuals to detach from the burden of guilt, which Islam addresses through mercy and repentance. Therefore, asking for God’s forgiveness might be a gateway to self-forgiveness.

Research often emphasizes that spirituality contributes to a higher level of self-forgiveness: people who feel close to God might admit their offenses freely and work on them eagerly (Davis et al., 2013; Hall et al., 2020). This also resonates with the results of the attachment studies, where secure attachment style was identified as a factor enabling individuals to forgive themselves (Eraslan Çapan, 2018). However, there is limited evidence on the role of spiritual attachment in self-forgiveness. In line with the theoretical background and empirical findings in related area, it can be inferred that it might be easier for individuals to forgive themselves when God is perceived and believed as an attachment figure who is compassionate toward His servants, has unconditional love for them, and excuses them for the wrongdoings they have committed. This positive image of God might encourage individuals to come clean, be honest and genuine with themselves and God for the change they have promised, and take action. Therefore, it is argued that spiritual attachment may be related to a better practice of self-forgiveness.

*H2*. Spiritual attachment has a positive direct effect on self-forgiveness: *value reorientation* (H2a) and *esteem restoration* (H2b).

Furthermore, research also suggests that self-forgiveness boosts wellbeing (Lim, 2015). By forgiving themselves, people might elude self-directed feelings of anger, hatred, and shame, and engage in the restoration of their esteem and values (Griffin et al., 2024). The mental change required by self-forgiveness requires individuals to look at themselves with compassion, build self-awareness, and reach a broader meaning of life (Webb et al., 2017). Therefore, the emotional and behavioral change followed by the evolution of this cognitive mindset might allow people to function and feel better. Especially, when combined with spirituality, self-forgiveness alleviates the negative mood and intention to self-harm (Nagra et al., 2016), hence promises an increase in mental health (Friedman et al., 2010; Romero et al., 2006). However, there is limited knowledge on the role of spiritual attachment in this interplay. In social sciences, it can be said that most of the time, there are some intriguing factors between psychological concepts. Therefore, self-forgiveness might act as a mediator in the relationship between spiritual attachment and flourishing. Spiritual attachment might indirectly relate to a better psychological state through its link with self-forgiveness.

*H3*. Self-forgiveness, *value reorientation* (H3a) and *esteem restoration* (H3b), has a positive direct effect on flourishing.

*H4*. Self-forgiveness, *value reorientation* (H4a) and *esteem restoration* (H4b), play a mediating role in the relationship between spiritual attachment and flourishing.

## Methods

### Sample

The present study recruited 311 (72.3%) females and 119 (27.7%) males, totaling 430 volunteer college students from 58 colleges and 18 faculties in Türkiye, using convenience sampling. The average age was 21.52 (3.49) for the total sample. All the participants identified themselves as Muslim, followers of Islam. Most of the sample has Turkish origin (*n* = 415, 96.5%), and a minor part (*n* = 15, 3.5%) has non-Turkish origin, mainly from the Middle East and Africa. Most of the participants were studying at the Faculty of Education (*n* = 148, 34.4%), followed by the Faculty of Health Science (*n* = 88, 20.5%) and the Faculty of Engineering (*n* = 52, 12.1%). Most of the sample are sophomores (*n* = 217, 50.4%). Most participants reported that the primary residential place is a province (*n* = 179, 41.6%) followed by a district (*n* = 107, 24.9%) and metropole cities such as Istanbul, Izmir and Ankara (*n* = 78, 18.1%). Lastly, the majority of the sample perceive themselves as middle-income (*n* = 370, 86.0%), and a very small part of the sample fit themselves in low-income (*n* = 35, 8.2%) and high-income status (*n* = 25, 5.8%). The demographic information is presented in Table 1.

**Table 1:** Demographic characteristics.

Variable	Group	*n*	Percent (%)
Sex	Female	311	72.3
	Male	119	27.7
Nationality	Turkish	415	96.5
	Non-Turkish	15	3.5
Collage grade	Freshman	79	18.4
	Sophomore	217	50.4
	Junior	64	14.9
	Senior	70	16.3
Primary residential place	Village/Town	66	15.4
	District	107	24.9
	Province	179	41.6
	Metropole city	78	18.1
Perceived socio-economic status	Low-income	35	8.2
	Middle-income	370	86.0
	High-income	25	5.8

*N* = 430.

### Materials

#### Demographic information form

The authors developed this form to obtain the demographic characteristics of the sample. It included questions regarding gender, age, nationality, college, college grade, faculty, primary residential place, perceived socioeconomic status, and psychiatric history.

#### Flourishing scale (FS)

The Turkish version of the Flourishing Scale (FS; Diener et al., 2009) was designed to evaluate individuals’ overall wellbeing and flourishing in life. The adaptation of this valuable tool was conducted by Telef (2013). The structure of the original FS was retained in the Turkish version. The Turkish version of the FS comprises eight items with a 7-point Likert-type scoring system (1–7; totally disagree to totally agree) under a single factor. The higher score illustrates higher flourishing. Sample items include “I lead a purposeful and meaningful life,” “I am engaged and interested in my daily activities,” and “I am competent and capable in the activities that are important to me.” The scale demonstrated evidence of good construct validity by Confirmatory Factor Analysis (CFA; CMIN/DF = 4.545, NFI = 0.94, RFI = 0.92, IFI = 0.95, CFI = 0.95, GFI = 0.96, SRMR = 0.04, RMSEA = 0.08) and internal consistency reliability (*α* = 0.96). The internal consistency of the scale was calculated as *α* = 0.855 in the present study.

#### Muslim spiritual attachment scale (M-SAS)

The Turkish version of the Muslim Spiritual Attachment Scale (M-SAS; Miner et al., 2017) aims to assess the attachment of individuals believing in Islam to God. The scale was adapted into Turkish by Yildiz et al. (2019). With some minor changes, the Turkish version of M-SAS comprises 15 items with a Likert-type scoring system (1–5; strongly disagree to strongly agree) under three subscales: Secure relationship (SR), positive model of self (PMS), and separation protest (SP). Sample items include “My confidence in God’s closeness and responsiveness encourages me to call on Him” (SR), “God’s love for me is unconditional” (PMS), and “I have cried out to God at times when He seems far away” (SP). Both subscale-based scores and the general sum of the scale can be calculated. Higher scores indicate a more secure attachment to God. The scale has evidence of good construct validity by CFA (CMIN/df = 2.57, NFI = 0.938, RFI = 0.923, IFI = 0.961, TLI = 0.952, CFI = 961, RMSEA = 07) and internal consistency (*α* = 0.92, 0.93, 0.83, and 0.82, respectively, for M-SAS total, SR, PMS, and SP) and split-half reliability (*r* = 0.82, 0.88, 82, and 0.72 respectively). The scale’s internal consistency was calculated as *α* = 0.975 in the present study.

#### Self-forgiveness dual-process scale (SFDPS)

The Turkish version of the Self-Forgiveness Dual-Process Scale (SFDPS; Griffin et al., 2018) aims to assess individuals’ self-forgiveness. The scale was adapted into Turkish by Kaya et al. (2023). The original factor structure of the scale was retained in the Turkish version. The scale comprises 10 items with a 7-point Likert-type scoring system (1–7; totally disagree to totally agree) under two factors (5 items each): value reorientation orientation (VRO) and esteem restoration (ERS). Sample items include “I regret that my past actions violated my values” (VRO) and “I still love myself even though I did wrong” (ERS). The scale has no total score; scores are calculated separately for each subscale. Higher scores suggest greater self-forgiveness. The scale exhibited evidence of good construct validity by CFA (CMIN/df = 2.24, NNFI = 95, CFI = 0.97, SRMR = 0.045, RMSEA = 0.063) and internal consistency (*α* = 0.76 and 0.87, respectively, for VRO and ERS) and split-half reliability (*r* = 0.71 and 83 respectively). The present study calculated the scale’s internal consistency as = 0.616 and 0.875 for VRO and ERS, respectively.

### Procedures

The necessary permissions for materials were obtained and compiled in an online questionnaire. The materials were distributed via social media accounts, instant messaging groups, and web pages. The data was collected by the participants who voluntarily enrolled in the study. Several inclusion criteria were applied; participants had to provide informed consent, be over 18 years of age, be enrolled in a college, identify as Muslim, and report no history of psychiatric conditions. The data was screened for these criteria; one case was excluded due to ineligibility based on educational status. No participants reported psychiatric conditions. After these controls, the data was transferred to SPSS software for data analysis.

### Data analysis

First and foremost, the dataset was checked for missing data and outliers and further examined for normality and multicollinearity (Field, 2013). The total scores were converted into standardized *z*-scores to identify outliers. The *z*-values ranging from −3 to +3 indicate that the data set had no sign of outliers (Tabachnick and Fidell, 2014). Therefore, the current dataset did not demonstrate any significant outliers. Subsequently, the skewness and kurtosis values were computed to assess normality. The results ranged from −2 to +2, indicating that the data followed a normal distribution (George and Mallery, 2019). Furthermore, the data was also checked for multicollinearity assumptions. Some criteria suggested in the literature include that the correlation between study variables should not exceed 0.90 (Field, 2013), the variance inflate factor (VIF) should be below 5, and the tolerance values should be higher than 0.20 (Hair et al., 2011, 2019). The estimated correlations, VIF, and tolerance values were *r* = between 0.249 and 0.462, VIF = 1.110 and 1.066, and tolerance = 0.901 and 0.93, respectively. Therefore, no critical evidence was found for multicollinearity in the dataset.

After these investigations, the dataset was deemed ready for analysis. The correlations were estimated with the Pearson correlation coefficient. The mediating role of self-forgiveness in the relationship between spiritual attachment and flourishing was tested using the PROCESS macro (Model 4) embedded in IBM SPSS software and in accordance with the guidelines provided by Hayes (2022).

### Bootstrapping procedure

The present study utilizes the bootstrapping procedure to test whether the indirect effect in the mediation model is significant. This approach, using the ordinary least squares (OLS) model and the bootstrap technique, is favored over the traditional three-step method due to its superior statistical power (Preacher and Hayes, 2008). A bootstrapping approach with 10,000 bootstrap samples was implemented within a 95% confidence interval to assess the statistical significance of the indirect effects. The absence of zero within the lower and upper bounds of the confidence interval indicates that the indirect effects are statistically significant (Hayes, 2009).

### Power analysis

Power analysis was conducted to estimate the minimum sample size required for the present study. The total sample size was determined to be a minimum of 107 individuals through an “*a priori* analysis” using G*Power 3.1 software for multiple regression analysis (Faul et al., 2009), with parameters set to two predictor variables, an alpha level of 0.05, a power ratio (1 − *β*) of 0.95, and a medium effect size (Balkin and Sheperis, 2011). The study’s sample size of 430 participants is sufficiently robust for the analysis. The power analysis results indicated a high effect size (*f*^2^ = 0.36) (Cohen, 1992; Faul et al., 2009).

### Findings

#### Preliminary findings

The preliminary analysis revealed that the study variables demonstrated low to high correlations. Flourishing found to have significant positive correlations with spiritual attachment (*r* = 0.416, *p* < 0.01), value reorientation (*r* = 0.349, *p* < 0.01), and esteem restoration (*r* = 0.462, *p* < 0.01). Similarly, spiritual attachment was positively correlated with value reorientation (*r* = 0.315, *p* < 0.01) and esteem restoration (*r* = 0.249, *p* < 0.01). The results are presented in Table 2.

**Table 2:** Preliminary findings.

Variable	2	3	4	Mean	SD	Skewness	Kurtosis
1. Flourishing	0.416**	0.349**	0.462**	20.277	7.878	−1.054	1.618
2. Spiritual attachment		0.315**	0.249**	59.386	14.959	−1.452	1.863
3. Value restoration orientation			0.257**	26.556	4.984	−0.775	0.957
4. Esteem restoration orientation				26.709	6.547	−0.932	0.730

*n* = 430, **p < 0.01, SD = standard deviation.

#### Mediation analysis

**Figure 2:**
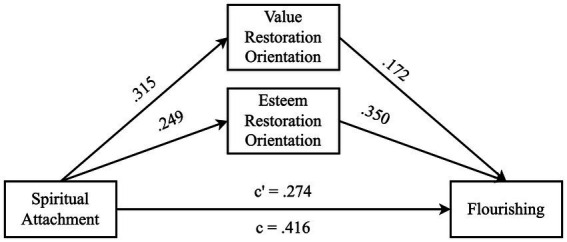
Results for the mediation model.

The mediation model was initially tested to examine the role of spiritual attachment in flourishing and the mediating role of self-forgiveness in the relationship between spiritual attachment and flourishing. The results are shown in Table 3 and Figure 2. First, the direct effect of spiritual attachment (path c) was investigated individually. The results showed that spiritual attachment had a significant positive effect on flourishing (path c, *β* = 0.416, *SE* = 0.023, *t*_1,428_ = 9.454, *p* < 0.001). In addition, spiritual attachment explained 17% of the variance in flourishing (*F*_1,428_ = 89.378, *p* < 0.001). Hence, the results provided robust evidence for Hypothesis H1.

**Table 3:** Results of mediation analysis.

Mediator	*a_n_b_n_*	95% CI		*a_n_*	*b_n_*	*c*	*c^1^*
		LL	UL				
Value restoration orientation	0.054	0.021	0.093	0.315**	0.172**	0.416**	0.274**
Esteem restoration orientation	0.087	0.040	0.143	0.249**	0.350**		

**p < 0.001, ab = estimated indirect effect, confidence intervals were generated with *n* = 10,000 bootstraps.

Subsequently, the direct effects of spiritual attachment on value and esteem restoration (paths a_1_ and a_2_) were investigated. The results showed that spiritual attachment had a significant positive effect on value reorientation (path a_1_, *β* = 0.315, *SE* = 0.015, *t*_1,428_ = 6.868, *p* < 0.001) and explained 10% of the variance (*F*_1,428_ = 47.172, *p* < 0.001). Similarly, the results demonstrated that spiritual attachment had a significant positive effect on esteem restoration (path a_2_, *β* = 0.249, *SE* = 0.020, *t*_1,428_ = 5.311, *p* < 0.001) and explained 6% of the variance (*F*_1,428_ = 28.205, *p* < 0.001). Therefore, Hypotheses H2a and H2b were supported.

Next, the direct effects of spiritual attachment, value reorientation and esteem restoration were examined within the same regression model. The results showed that spiritual attachment (path c^1^, *β* = 0.274, *SE* = 0.022, *t*_3,426_ = 6.484, *p* < 0.001), value reorientation (path b_1_, *β* = 0.172, *SE* = 0.067, *t*_3,426_ = 4.064, *p* < 0.001), and esteem restoration (path b_2_, *β* = 0.350, *SE* = 0.050, *t*_3,426_ = 8.411, *p* < 0.001) had significant positive effects on flourishing. The results also revealed that all three IVs explained 34% of the variance (*F*_3,426_ = 71.775, *p* < 0.001). The effect size for the multiple regression based on all IVs was estimated as *f*^2^ = 0.506. Cohen (2016) proposed that the estimate of 0.35 is a large effect size. These results indicated that Hypotheses H3a and H3b were supported.

Lastly, the indirect effects were examined (paths a_1_b_1_ and a_2_b_2_). The indirect effect of spiritual attachment on flourishing through value reorientation and esteem restoration was significant (*β* = 0.141, *SE* = 0.034, 95% CI [LL = 0.079, UL = 0.213]). When the individual indirect paths were examined, a similar picture was seen. The individual indirect effects through value reorientation (a_1_b_1_, *β* = 0.054, *SE* = 0.018, 95% CI [LL = 0.021, UL = 0.093]) and esteem restoration (a_2_b_2_, *β* = 0.087, *SE* = 0.026, 95% CI [LL = 0.040, UL = 0.143]) were found significant. Therefore, it can be said that Hypotheses H4a and H4b were supported.

The results illustrated that spiritual attachment contributed to the flourishing of college students both directly and indirectly through self-forgiveness. Both dimensions of self-forgiveness contributed to flourishing and served as mediators between spiritual attachment and flourishing. This suggests that part of the effect of spiritual attachment may operate through individuals’ capacity for self-forgiveness.

## Discussion

The present study promises useful information of the role of spiritual attachment and self-forgiveness on flourishing among college students. First, it was found that the spiritual attachments contribute to better flourishing. It might improve overall wellbeing by various means. With more secure attachment to God, people might discover meaning and purpose of life (Panzini et al., 2017). It defines a quality of a good life and provides a sense of direction. It also makes people more emotionally resilient in the face of challenges and adverse events (Jones et al., 2016), as the ultimate source of power, God, is watching for them and there might be a higher meaning in the struggles. In addition, attachment to God might enable individuals to do the deeds asked by Him, including the ones that enhances social connections: be kind to people, respect the parents, help people in need, do not lie to them, forgive the wrongdoings of others, and so on. This might create a social agenda for individuals (Saslow et al., 2013) and improve social support systems which contributes to a better mental health (Salsman et al., 2005).

The second hypothesis was supported by the data and provided evidence on the positive effect of spiritual attachment on self-forgiveness. To understand what this result means in the target population of the present study, some might need to understand the concept of forgiveness in Islam. In many places in The Holy Quran (2019), God warns the sinners about the impending day of judgment, where intense torments were prepared for them. However, at the same time, God often underline that He is compassionate for His servants, forgives them very much, and also encourages them to forgive each other in various contexts:

“Let not those among you who are endued with grace and amplitude of means resolve by oath against helping their kinsmen, those in want, and those who have left their homes in Allah’s cause: Let them forgive and overlook! Do you not wish that Allah should forgive you? Allah is the Most Forgiving, the Most Merciful.” (The Holy Quran, 2019; Surah An-Nûr, Verse 22, pp. 361).

Yet researchers argue that the concept of self-forgiveness is incompatible with Muslim commitments, which rest upon the fact that only God has the authority to forgive a sin; therefore, sinners do not have any power to forgive themselves (Ghorbani et al., 2017). Still, in the holy messages, it is pointed out that Allah will forgive people who feel sorry for their offenses and promise not to do so. This commitment is an internal change that individuals are genuinely regretful of. Therefore, it is the process already outlined by self-forgiveness efforts: acknowledge the wrongdoings, accept the imperfect nature of self, and change attitudes toward them. Khan (2024) draws attention to this similarity and emphasizes that the underlying mechanism of self-forgiveness aligns with the Islamic concepts and procedures. He claims that the common stages involve being regretful about the offenses, repenting, taking responsibility, and releasing negative feelings and thoughts. This spiritual perspective often stresses the role of repentance (Tawbah) on the path of spiritual healing (Toscano, 2022). There is a key nuance that the repentance requires an internal change. It also includes behavioral change where individuals are expected to show appropriate behaviors and atone for the damages they have caused (Rassool, 2021).

The critical difference here might be the perception of Allah. If believers perceive God as a tormentor, then their internal working models will be built on the idea that “being with someone” comes with tremendous pain. Hence, it might result in an insecure attachment to God. In parallel with this negative image of God, people might internalize the standards of punishment. This might cause a conception of a mindset that prevents individuals from thinking positively about themselves and the deeds they have done. On the other hand, God as a positive attachment figure might encourage individuals to self-forgiveness. In Islamic perspective, there is a positive image of Allah, because He is the one with the Mercy (Rahmah) and He often encourages His servants to self-correct and repent (Tawbah) for their offenses (Baharudin et al., 2019). A simple example from Bowlby’s (1969) and Ainsworth’s (1978) perspective can illuminate this fact: A child who sees his/her parents forgiving, compassionate, and full of unconditional love, might feel secure to confess his/her misbehaviors, built self-awareness with the help of their guidance, and restore his/her perception of self. A child who is fearfully attached to his/her parents might not even get close to admitting the wrongdoings, where change might not even spark. In support of this, Liao and Wei (2015) concluded that self-forgiveness might be a transformative cognitive process, where forgiveness of self might help individuals transform the harsh self-evaluations stemming from negative early experiences that resulted in insecure attachment into neutral or even positive ones, which are linked to fewer depressive symptoms. Similarly, a recent study conducted by Cornish et al. (2024) found that self-forgiveness mediated the relationship between attachment anxiety (which might be seen as a function of insecure attachment) and personal wellbeing.

The study also frames the evidence that self-forgiveness contributes to better flourishing. It might contribute to a better state of mind in various ways. By forgiving themselves, people might alleviate the adverse effects of self-directed hatred, guilt, and shame, and redirect their energy to empower personal growth and change (Ciminli and Kaya, 2023; Eroglu et al., 2022; Fisher and Exline, 2010). Carrying self-blame and guilt often causes anxiety, depression, and low self-esteem (Zahn et al., 2015). Therefore, self-forgiveness might help alleviate these negative states by promoting self-compassion and acceptance. Self-forgiveness enhances coping self-efficacy, resilience, and mindfulness (Kaya and Odacı, 2024) by encouraging individuals to learn from their mistakes rather than dwell on them. It allows for a shift in mindset from self-punishment to personal growth. When individuals forgive themselves, they see their mistakes as opportunities for learning, which helps them bounce back more quickly from setbacks and challenges. This growth mindset is critical to flourishing because it promotes adaptability and continuous improvement. Self-forgiveness is associated with emotional regulation and resilience, which individuals might benefit from to increase positive emotional states and eliminate negative ones (Malakcıoğlu, 2022). Therefore, the presence of positive emotions and the absence of negative emotions are fundamental elements of the hedonic perspective of human wellbeing and flourishing (Ryan and Deci, 2001). Also, when individuals forgive themselves, they are more likely to extend forgiveness and understanding to others (Woodyatt and Wenzel, 2013). Self-forgiveness encourages empathy, as people who are kind to themselves are often kinder to others, which allows for healthy relationships. Therefore, healthy relationships are a cornerstone of flourishing, contributing to a sense of belonging and social support (Diener et al., 2010; Keyes, 2015).

Lastly, the present study revealed that self-forgiveness mediates between spiritual attachment and flourishing. By securely attaching to God, internal working models allow individuals to relate to others positively, including self (Bowlby, 1969). Individuals who believe that God is merciful and encourage them to be better people might have a favorable attitude toward themselves. This might trigger an internal change after committing offenses that bring self-compassion, acceptance, awareness, and personal growth. This mindset might help individuals break the negative attributions to self, eliminate disruptive emotional problems, and act proactively. In this way, individuals might gain better functionality and mental health. In line with this, Samen et al. (2024) found that early childhood maltreatment is strongly linked to problematic relationships with the father, and the two variables also predict later maladaptive schemas. On the other hand, self-forgiveness emerged as a protective factor in their study. The authors recommend that interventions promoting healthy child–parent relationships and self-forgiveness can help individuals have better wellbeing in the future. Similarly, individuals’ relationships with God develop from childhood to adulthood, where their spiritual experiences and significant others’ teachings on spiritual topics might shape their attachment to God. Secure attachment to God might be linked to adaptive schemas and cognitive functioning, self-forgiveness, and flourishing. In the Islamic perspective, they built a bond with God and feel attached to Him as the bond extends through sharia, tariqa, ma’rifa, and haqiqa (Özdemir, 2021). This bond might provide a direction to the life, where individuals repent (Tawbah) and seek forgiveness from God (Istighfar), and feel inner peace (Karakaş and Geçimli, 2017; Rassool, 2024).

## Limitations and future directions

Readers need to consider a few of the present study’s limitations while interpreting its results. First and foremost, the study was conducted on a sample of all-Muslim college students, which might hinder the generalizability of the findings to non-Muslim samples. Although the function and experience of religion might share a common root, especially in Abrahamic religions, it is essential to keep in mind cultural and religious differences. Therefore, future studies should focus on cross-cultural comparisons regarding the role of spiritual attachment and self-forgiveness in flourishing.

Second, the present study’s sample was recruited through convenience sampling. Convenience sampling has certain limitations in terms of the generalizability of the results. To address this issue, the demographic characteristics of the participants were reported extensively. However, since women, students in the faculty of education, and students who perceive themselves as belonging to middle-income families were overrepresented in the current sample, readers should evaluate the findings with these characteristics in mind.

Third, the value reorientation subscale in the data collection tools showed a slightly weak internal consistency reliability. Item-total statistics were examined to specify whether the low coefficient resulted from an incompatible item in the current sample. However, it was found that deletion of any of the items did not improve the alpha. Therefore, this might be due to a more general reason. One possible explanation is that the individuals’ experiences might be very diverse regarding actions conflicting with their values. This might create discrepancies between individual responses to the items, leading to a drop in internal consistency. Another explanation might be that the individuals’ previous actions conflict with their past values. Therefore, the values they rely on can be changed. This might also result in variability in the responses of Turkish college students. As emerging adults, their identity development is still progressing: they actively evaluate their values and make some adjustments. The low internal consistency reliability of the VRO might have been reflected in the results, where the VRO demonstrated lower coefficients than the ERO subscale in predicting flourishing. Thus, future studies employing a qualitative research design might provide a better understanding of the value reorientation of Turkish college students.

Fourth, the mediational models tested with cross-sectional data cannot provide information on causal interactions. Therefore, the results of the present study should be considered preliminary due to the lack of experimental or longitudinal procedures. Despite this shortcoming, the present study is grounded on a solid theoretical and evidence-derived basis. Based on the current study, researchers might infer possible causal directions and design a longitudinal study to explore causal relationships between emerging adults’ spiritual attachment, self-forgiveness, and flourishing.

Lastly, on sensitive topics like religion, participants might respond to items in a socially desirable way. Self-report-based instruments generally suffer from this problem. These biases might affect the results and present an “ideal” image that is not entirely accurate. Hence, it would be beneficial for researchers to consider this and provide clarifications for potential participants before data collection.

The present findings can also provide some practical implications for university counseling services. Students who exhibit low levels of flourishing and high levels of psychological symptoms, such as stress, anxiety, and depression, might experience problems in forgiving themselves because of their previous wrongdoings. These acts might include elements of shame and guilt stemming from a confrontation with personal values, and especially religiously influenced standards. It would be helpful for practitioners to explore the spiritual attachment or the bond between the person and God. Knowing individuals’ history with God—how the attachment between the individual and the divine has developed—and how they perceive Him, might help practitioners better understand the underlying difficulties in self-forgiveness. Therefore, helping individuals restore their spiritual connection might support them mentally.

## Conclusion

The present study offers valuable insights into the role of spiritual attachment in the mental health of college students. Feeling attached to God enables individuals to flourish better in life. In Islam, like other religions, God has supreme power. As His servants, people ask for God’s love, compassion, forgiveness, endowment, and protection. By being attached to God, individuals feel ease and relief as if the help of the Greatest is with them. This might encourage individuals to be actively involved in life challenges and cope effectively. It might foster a positive mindset where individuals see life as a better place. These cognitive, emotional, and behavioral reflections of spiritual attachment might promote better wellbeing. Also, the present study concluded that self-forgiveness might interact with the interplay between spiritual attachment and flourishing. By believing that one possesses God’s unconditional love and forgiveness, people might forgive themselves for their wrongdoings in the past. This might shatter the barriers individuals face when engaging in life, as people experience remorse, guilt, and anxiety for their offenses, do not see any chance to get through these situations, believe that there is no way for redemption, and often withdraw themselves from confronting them. These results might be helpful for practitioners in the field, where college students might be supported in their spiritual dilemmas and past experiences to promote better flourishing.

## Data Availability

The raw data supporting the conclusions of this article will be made available by the authors without undue reservation.
